# The role of music performance anxiety in musical training: four personal histories

**DOI:** 10.3389/fpsyg.2025.1515970

**Published:** 2025-02-10

**Authors:** Oscar Casanova, María Elena Riaño, Francisco Javier Zarza-Alzugaray, Santos Orejudo

**Affiliations:** ^1^Department of Musical, Plastic and Body Expression, University of Zaragoza, Zaragoza, Spain; ^2^Department of Education, University of Cantabria, Santander, Spain; ^3^Department of Psychology and Sociology, University of Zaragoza, Zaragoza, Spain

**Keywords:** music education, musical performance anxiety, life histories, conservatory students, thoughts of abandonment, qualitative methodology

## Abstract

Musical Performance Anxiety (MPA) is one of the major obstacles many musicians face in the course of their training and careers. Few studies have approached this construct using a qualitative methodology. To apply such an approach, we gathered testimonials of life histories from four musicians – two pianists, a violinist, and a cellist – through semi-structured interviews. With an average age of 25, they had all studied classical music for at least 10 years. We subjected the protocols of those interviews to a phase and categorization process based on Interpretative Phenomenological Analysis. The first years of training were those when our interviewees recalled experiencing the greatest enjoyment in music-making: positive elements included family support, ensemble playing, and initial encounters with non-classical repertoire and improvisation. However, as our interviewees progressed through their academic courses and improved in terms of mastery of their instrument, they began to experience situations of anxiety. Physical symptoms appeared, regularly associated with situations such as examinations and auditions in front of a jury. Two of four respondents decided to terminate their music training before entering university level. To deal with symptoms of anxiety, several approaches were pursued: visualization, cognitive analysis, and self-medication. However, to mitigate MPA, our informants generally recommend more rational strategies: a realistic focus on objectives coupled with reinforcement of self-esteem. Institutions of musical learning can help students cope with this disorder by encouraging musical creativity and selecting educators who apply empathetic teaching styles well-adapted to their pupils’ needs. In the future, this type of qualitative research can be expanded to a greater number of informants with more diverse characteristics. The qualitative approach will help us to better understand the MPA phenomenon.

## 1 Introduction

Music performance anxiety (MPA), commonly known as stage fright, is a psychological condition that affects musicians when they have to perform in public. It is a physico-emotional response that emerges when individuals experience fear and anxiety associated with the possibility of facing an audience or having to interpret a musical work in a situation where they feel they are being evaluated (Herman and Clark, 2023; Kenny, 2011). Such anxiety can affect musicians at all levels, from amateurs to experienced professionals. It can have grave consequences for an artist’s musical career. A series of studies have assessed that the prevalence of MPA among musicians ranges between 16.5 and 60.0% (Fernholz et al., 2019).

MPA can appear in several forms. Certain musicians experience physical symptoms such as excessive sweating, tremors, palpitations, muscle tension, nausea, and diarrhea. Others may have recurring negative thoughts, such as worrying about making mistakes, fear of rejection, and lack of confidence in their musical ability. These symptoms can interfere with a musician’s performance and diminish their ability to play or sing at their best (Herman and Clark, 2023; Kenny, 2011; Osborne and Kirsner, 2022). According to Papageorgi et al. (2007), anxiety in the context of a musical performance is a process that possesses an explicitly temporal dimension (before, during, and after the performance). It consists of a series of probable events or processes that tend to occur when a musician knows they will take part in a particular performance situation. MPA can give rise to certain maladaptive or adaptive forms of anxiety.

Recent studies on MPA in music students (Casanova et al., 2018; Orejudo et al., 2021; Zarza-Alzugaray et al., 2018, 2020) have indicated differences among MPA levels according to the type of instrument, academic year, age of musical training onset, the type of educational institution, and the eventual level of substance abuse. Those studies also incorporated further constructs, including self-efficacy and social support. Furthermore, different studies (Cupido, 2018; Dobos et al., 2019; Liston et al., 2003; Patston and Osborne, 2016) have linked MPA with personality traits, including perfectionism.

In some cases, the grave consequences of MPA decide whether a musician will pursue a promising career or not. Artists suffering from this disorder may feel unable to perform in public, limiting their ability to promote their music and generate income. In addition, MPA can significantly reduce the quality of a musician’s performance. This, in turn, will negatively affect their reputation and reduce their ability to secure new contracts.

Fortunately, there are several ways of tackling MPA and minimizing its adverse effects (Burin and Osório, 2016, 2017; Herman and Clark, 2023; Kenny, 2005, 2011, 2016).

Osborne and Kirsner (2022) highlight the following main therapeutic approaches developed for MPA: cognitive therapy, which attempts to change distorted thought patterns; behavioral therapies, which focus on changing dysfunctional behaviors that occur when people become anxious; cognitive behavioral-therapy (CBT), which combines cognitive and behavioral approaches; acceptance and commitment therapy, which encourages mindfulness and acceptance of emotional distress; psychodynamic therapies, which view MPA as a symptom of a musician’s internal conflict; coaching and performance practice approaches, which include pre-performance preparation, the use of imagery (in which performers imagine certain scenarios and aspects of the performance), and mastery and performance goals (with specific, realistic and timed objectives); pharmacological interventions, mainly involving the use of beta-blockers or anxiolytics; and other therapeutic approaches, such as the Alexander Technique.

Thus, a variety of practical strategies may be useful in each individual case and should ideally be addressed during the musician’s training (Osborne and Kirsner, 2022). These may include strategies for managing somatic arousal, cognitions or behavior.

One of the main behavioral strategies consists of ensuring adequate preparation and rehearsal. Musicians who feel confident in their ability to play a piece of music may feel more at ease by the time they face an audience. Practicing regularly and rehearsing in environments that emulate concert situations can help reduce anxiety.

Another effective behavioral strategy can be found in visualization, which is a form of mental rehearsal, i.e., imagining a successful performance prior to the real one. Musicians can visualize themselves performing in front of members of an audience who are sitting in silence and listening attentively. They can also imagine themselves enjoying the experience while remaining secure and confident in their capacity to play. Visualization techniques can help musicians reduce stage anxiety and increase their self-confidence.

Prior to performance, relaxation techniques such as deep breathing, yoga, and meditation can also be helpful in tackling musical stage fright.

Building on these behavioral methods, which include relaxation techniques with cognitive strategies, is CBT. It is effective in helping musicians focus on identifying negative thoughts that contribute to anxiety, while encouraging them, instead, to replace them with more positive, realistic thoughts. Regarding gradual exposure techniques, for example, CBT for MPA involves performers moving through a hierarchy of increasingly aversive performance situations only when they feel comfortable performing at the earlier level.

Most studies in the field of MPA have adopted a clinical approach (Barros et al., 2022; Burin and Osório, 2016, 2017; Fernholz et al., 2019; Herman and Clark, 2023). More often than not, the chosen methodology has been quantitative (Papageorgi and Welch, 2020). The literature review indicated that more qualitative studies would be needed to complement the findings obtained through quantitative methods. Few studies have used qualitative methodologies to explore further relevant aspects regarding MPA. Moreover, few of them have studied MPA from the perspective of the life histories of people who have suffered from the disorder and who either abandoned their music studies or who, on the other hand, gathered up the courage to persevere by pursuing a professional music career. Among the qualitative studies in the field of MPA, two that conducted a phenomenological analysis (Interpretative Phenomenological Analysis, IPA) stand out: on the one hand, Papageorgi and Welch (2020) interviewed four professional musicians; on the other hand, Robinson and Nigbur (2018) interviewed four university-level music students.

These two studies use the IPA to shed further light on the lived experience of performance anxiety and suggest that the IPA is a useful research tool to facilitate our understanding of this subjective experience. The first of these studies focused on two males and two female musicians performing as part of an ensemble (orchestra, band), so solo musicians, “who may well have different (more intense) experiences” (Papageorgi and Welch, 2020, p. 10), were not analyzed. In the second study, four students (three female and one male) were selected from the final year of the music department at the same university. These were “non-professional music undergraduate for whom feedback on performance is an integral part of the course” (Robinson and Nigbur, 2018, p. 18) and the “interviews covered both solo and choral performance” (p. 19). However, participants in both studies did not express a firm intention to abandon their studies or their music careers.

It could thus be pertinent for other research teams to analyze the information provided by further interview respondents who have suffered from MPA, studying their experience and noting the eventual coping mechanisms they have developed. People who have had recurrent thoughts of abandonment, and have even materialized it. “In the scientific literature it is difficult to find studies based on the determinants of the abandonment of musical education” (Orejudo et al., 2021, p. 469). This, in turn, could help to improve the process of learning and teaching music. The overall objective of our study was thus to gain insight by exploring and analyzing the subjective lived experience of MPA in musicians who have suffered from it.

## 2 Studying the issue

MPA has been the subject of much research, looking at its prevalence, risk factors, interventions and the lived experiences of musicians. The main findings and relationships identified in the literature review on this topic are presented below.

In terms of prevalence and associated factors, the literature review by Burin and Osório (2016, 2017) shows that MPA is common among musicians from diverse cultures and levels of training. Cognitive, behavioral and physiological factors, as well as biological and psychological predispositions, are relevant to its etiology. Predictors of MPA related to the individual, task and musical situation have been identified. The review by Fernholz et al. (2019) highlights that women are more affected than men, and that older musicians have a lower incidence of MPA.

Burin and Osório (2016) identified several effective interventions for MPA, including CBT, biofeedback and yoga. The review by Fernholz et al. (2019), which used standardized assessment tools for the first time, highlighted the effectiveness of CBT and *β*-blockers as beneficial treatments. However, in their review, Herman and Clark (2023) questioned the limited effectiveness of current approaches and suggested the need for new treatments.

Coping strategies include breathing and relaxation techniques and increased musical practice (Burin and Osório, 2017). Tahirbegi’s (2022) study highlights the importance of teacher and peer coaching and support in managing MPA, and links self-efficacy to experiences of reduced anxiety.

Ananias de Lima et al. (2024) address MPA in adolescent musicians and propose that the development of effective prevention and intervention strategies can promote emotional resilience and well-being in this population. This approach suggests that early attention and ongoing support are critical to addressing the challenges of MPA.

Regarding the subjective experiences and perceptions of musicians, qualitative research (Bober, 2019; Clark et al., 2007; Cupido, 2018; Robinson and Nigbur, 2018) reveals that MPA is related to audience perception (audience concern), trust issues and musical identity. Also, preparation and a positive mind-set are linked to successful performances. MPA is experienced differently depending on the context and type of music. Self-induced pressure and personal standards are identified as triggers for MPA.

Burland and Davidson (2002) analyzed the reasons for pursuing or not pursuing a professional career. The centrality of music to self-concept and positive experiences (with others and within music education institutions) appear to be key factors in successful transition from training to professional life. In the study by Orejudo et al. (2021) with a sample of 463 university students from different instrumental specialities in Spain, 19% had considered abandoning their music studies in relation to their self-reported MPA level.

Following their literature review, Barros et al. (2022) highlight the diversity of contexts and the lack of qualitative and longitudinal studies on MPA. They note the diversity of approaches and suggest that future research should include different methodologies to better understand this complex experience.

In summary, MPA is a multifaceted phenomenon that affects musicians of different ages and genders. Interventions should be personalized and take into account individual and contextual factors to be effective. Future research should focus on innovative approaches and the exploration of subjective experiences to improve the management of MPA.

## 3 Methodology

This study is part of a larger research project. Among other aspects, MPA is studied quantitatively among students of several Spanish music conservatories at elementary, professional (*Grado Profesional* in Spain) and higher (university) levels, and several specific interventions are also carried out to deal with MPA with some groups of students at these levels. Qualitatively, information is collected from teachers through focus groups and, through different qualitative strategies, relevant information is obtained on specific aspects related to the different agents (teachers, parents, students and professional musicians).

In accordance with this study’s objectives, we applied a qualitative methodology with the purpose of gathering significant information from reports of real-life situations. Our ultimate goal was to describe reality as it is apprehended by subjects within the context in which reality is constructed (Denzin and Lincoln, 2005; Flick, 2014, 2015; Gibbs, 2012). Our study thus sought to “unpick how people construct the world around them, what they are doing or what is happening to them in terms that are meaningful and that offer rich insight” (Flick, 2015, p. 11).

As an instrument for gathering the informants’ perceptions, we selected the semi-structured interview, a tool that allows researchers to obtain valuable data and identify interesting areas and topics that can arise during the interview process (Blaxter et al., 2008; Kvale, 2011).

Four semi-structured interviews were conducted with people relevant to the study. We were looking for people who had studied professional music education and had decided not to continue their studies, or who, despite having problems with the MPA and considering dropping out, had decided to continue. The informants were recommended by their former teachers. Although we selected them according to availability, we attempted to ensure the greatest possible variety among participants (Miles et al., 2013). Our informants were: a female violinist, a male cellist, a female pianist, and a male pianist. The mean age was 25. Two of our interviewees had finished their university-level music studies by obtaining a diploma; the two others had decided not to attend music university once they had finished conservatory (professional level in Spain). They had all studied classical music for at least 10 years, and their instruments were highly soloist. None of the participants received any medication or treatment for MPA during their studies.

Ethical approval for this research was obtained from the University of Zaragoza in compliance with the conditions required for this type of study. Our participants collaborated on a voluntary basis. After we explained our study’s goals in detail and answered all their queries, they signed an informed consent form. We asked for their permission to record the interviews, ensuring anonymity and confidentiality. Protection of privacy was ensured by omitting any detail that might make any participant identifiable.

Prior to the interview, we asked our four interviewees to write a personal history of their experience with MPA; the aim was to confirm that the selected participants had experienced high levels of MPA and had thoughts of abandonment. Once we had received those documents, we set an appointment date for the face-to-face interviews. To conduct the latter, we elaborated an interview guide containing the following main themes: (1) Personal history related to music and situations of music anxiety. (2) Factors of musical anxiety and ways of coping with them. (3) Approach to abandoning music studies.

The final script was developed after an earlier pilot experience with a person not involved in this study. Our interview guide contained precise questions. However, we only used it as a guide, allowing for a certain amount of flexibility as the interview progressed. The interviews lasted between 60 and 80 min. They were recorded on both audio and video.

We then transcribed and analyzed the interviews following the main analytical principles of IPA, i.e., Interpretative Phenomenological Analysis (Eatough and Smith, 2017; Smith and Osborn, 2015; Smith et al., 2009), a method designed to produce detailed examinations of individuals’ lived experiences described in their terms. The analytical procedure consisted of the following steps (1) Reading the transcript of the first interview several times and noting the most interesting points of discussion. (2) Reviewing the transcript and transforming the initial notes and ideas into more specific themes and phrases. (3) Narrow down the data further by making connections between the preliminary themes and grouping them accordingly. (4) Select descriptive headings for general themes to capture their conceptual nature. (5) Repeat steps 1–4 for all other interview transcripts. (6) Identify similarities and differences between the themes derived from the different interviews. (7) Organise the themes and sub-themes from the interviews. (8) Select representative examples that capture the essence of each theme and sub-theme. From this analytical process emerged the themes that appear in the results section. The original texts of the participants, translated from Spanish, are presented in this article.

## 4 Results

This section presents our results according to the found themes, illustrated with literal quotes from the four interviews.

### 4.1 Theme 1: personal performance history

The four musicians described situations of enjoyment and situations of anxiety associated with their musical and instrumental training throughout their personal history. The majority of instances of enjoyment were associated with their first musical experience in early childhood. Families played a key role in their children’s musical initiation. As the cellist points out (henceforth named Vc): “When I was a boy, I loved to hear music, and my parents noted that I liked it. […] They gave me piano lessons at home for a year.” The male pianist (henceforth referred to as P1) remembered the time when his parents gave him a musical toy as a present: “When I was five or so, they gave me a pocket keyboard you had to play using your fingernail. […] On that keyboard, I used to pick out the tunes from television advertisements. I loved it.” The two women (hereafter named “Vn” for the violinist and “P2” for the male pianist) started their instrumental training in music schools. All four participants affirmed that it was during those first years of training, including music school, where they had the majority of gratifying experiences in class, recitals, and auditions. “Yes, [it was] in the first years: first, second, and third year of music. That is perhaps where I had the most enjoyment.” (Vc); “When I was a little girl, I do not remember having any experience of anxiety. Perhaps one is not aware; you see it as a game” (P2).

Further positive situations arose when the subjects played music in a group that established an emotional connection among its members, as this interviewee recalls:

I have many lovely memories of playing, but one which comes particularly to mind because I think it was the first among my dearest memories, so to speak. What it was… I attended a specialization course when I was fourteen or so […] Fourteen or fifteen, I guess. In that course, we took instrument lessons, then played in several different formations. I played with a quartet; I believe we were all girls. They were somewhat older than me. In the rehearsals – an intense week – we would work a lot on the same piece, then perform it in front of the other [students]. In those rehearsals, I repeat, I wasn’t very used to playing with others in a group; until then, all my music lessons had been private. These rehearsals were based on the idea, “Let us make some music together.” I remember we tended to somewhat imitate our teachers, who also formed a string quartet. I do not know, I think I laughed a lot. It was my first time playing with others instead of playing alone. All the pressure disappeared. I just had a good time, really a great time. Because we would laugh together, in the midst of the… while we were playing; we made inside jokes, because… because we were under no type of pressure. All there was, was this “Hey, that sounds really good; let us enjoy it.” And yes, I remember there was definitely a “before” and an “after.” I ran back home and said, “Dad, you cannot imagine what a good time I had playing music [there].” [laughs] (Vn).

The same informant recalls a further moment in duet, playing non-classical repertoire. It turned out to be fun for her thanks to her emotional involvement with her colleague:

I had another [positive] one in [university-level] music academy when I devised my graduation project and played with a [female] friend. We spent a year playing a repertoire made up more of tangos and traditional [popular] music. That was a year of intense work, but also when we took up a series of pieces and made them our own, right? Then we made our own versions, and that was a moment when we felt really moved, right? We were exploring how to play those pieces, changing small details and such, and I remember having a great time. In those two positive experiences [adolescence, then university level], I think there was some emotional involvement with other people and an approach that did not just involve making sure that the notes were right; rather, something more than that. (Vn).

As the interviewees progressed through their academic courses and improved in terms of mastery of their instrument, they began to experience situations of anxiety. All four informants reported experiencing physical symptoms in moments of anxiety, such as trembling hands and feet, abdominal pain, or sweating. Such situations were frequently associated with conservatory entrance exams, examinations, and auditions:

When I was still a kid, I would just enjoy it. But I tell you for sure, it’s when that additional factor appeared – the need to gain entry into conservatory to be able to continue – that anxiety gradually started to creep in. [From then on,] my head was more intent on doing everything correctly instead of enjoying the music. So, at that point, indeed, I felt an anxiety, a nervousness that ultimately did not allow me to achieve what I simply intended. Physically, I had a trembling in my hands and elsewhere, but I think it was more psychological than anything else. (P1).

My hand started to tremble – something that had never happened to me before – and I was sweating. (Vn).

It happens to me particularly in instrument exams, either in cello [main instrument] or in piano as a second instrument. Or it appears in solo auditions… given that, long before the recital, I’m already thinking about the fact that I’m going to have to pass an audition, and in that sense, it makes me feel quite uneasy. […] Normally, [what happens is that] my belly aches a lot, and I get pretty nervous. Most of all, my hands tremble when I climb to the stage. When your hands tremble while playing an instrument like mine, it becomes easily noticeable, and the audience can tell. I remain nervous from beginning to end [of the performance], and that tends to demotivate me a great deal – as it’s pretty different just to play it at home, thinking that’s how it will sound in concert, but then you arrive [on stage], and you do not achieve the result you expected, and that demotivates one quite a bit. (Vc).

In fact, one of our interviewees recalled that they did not present themselves to an audition due to the anxiety they felt in imagining how they would have to go out on stage to play in front of others.

My [right] foot was my greatest worry, since I have to control the pedal, the pedals. And my foot trembled like you would not believe […] the hands […]. Physiologically, everything inside me is in uproar: the digestive system, particularly the gut […]. So, 1 week before [the event], I almost could not eat anything […]. Particularly at mealtime, I would stop to think, I do not know why […] My stomach just closed, I frequently went to the bathroom, I would sweat […], thinking about the moments just before [the performance]; I even thought to myself: I just hope this all ends. I hope they call me from the conservatory [to tell me that the building is] burning to the ground; hopefully, I’ll have some kind of injury. I admit that on occasion, I told them I had fallen seriously ill, and I did not go to play because I was simply overwhelmed by the situation. (P2).

Conversely, whenever these musicians did not feel they were being judged, their recollections of moments of enjoyment were evident:

[It was] in a piano competition, in which the winners – in this case, I was the winner – had to play in the final gala. So, at that moment, nobody was evaluating me, judging me... I mean, of course we were still being judged; the audience never stops being the judge somehow, but nobody was evaluating me anymore, it did not matter how I played. I simply had to stick to enjoying a piece I had studied very well and played so many times. Then I went out, I played, I enjoyed, I failed, and it did not matter a thing to me. That was it. So, I was thoroughly satisfied (P1).

### 4.2 Theme 2: critical moments

The critical moments containing the greatest difficulties for our four musician informants occurred in advanced specialization stages. In two cases, these were entrance exams to university-level music academies; in a further case, an entrance exam to secondary-level music school, along with auditions pertaining to the final academic years in another case. Anxiety was triggered by the fear of being judged by juries and by the possibility of seeing oneself compared with peers:

When I went to Barcelona, I must have been about 19 or 20; I have the worst memories because I started to feel more judged there... When I was a student, I had an audition in front of all my classmates and in front of three professors, and there I remember that I blocked myself; I went blank. No, I wasn’t able to... I made it through, but not at the level I was used to. I finished as best I could and went home. I do not remember whether I went home to cry, but it was terrible; it left a bad impression on me. (Vn).

It was at the age of 20, when I entered Superior [university-level] Conservatory, that I noticed a radical change in [my] stage anxiety. The goals were not so easy, the competition, well, let us call it “competition,” the classmates... It wasn’t really competition at all... [Competition] is when you have a real comparison because the evaluation is actually quite subjective. And that evaluation has an impact on the way you judge yourself throughout the exam. Sure, five people in front of you [are able to] play [at a certain level], and you come along – and I, for one, inevitably make a comparison, even if it’s subjective, and at any rate, they will be making a comparison when they evaluate me. And the teachers! In whichever audition, in front of whichever jury, in whatever audition or recital, you really did not care exclusively about what one teacher might think, but about the entire jury… Just the thought of realizing they were there, and anxiety was [already] present. (P1).

When I was admitted to [secondary level] music academy, that’s a point where one would suppose that you can already look back on a certain amount of experience playing in auditions. You would think that once you have reached that point, you will have to be able to control such things. But in my fourth year of [secondary level] music academy, I still did not know how to be calm. I was pretty nervous: my hands would tremble, and I could not coax a good sound from the cello … since to play the cello, you cannot be tense in your right hand or your left. But I was, thus, no way… the sound I got out of the cello wasn’t pretty at all. I sounded as if I was at a level several grades below mine. (Vc).

At one point in the last years, I was the oldest. I was twenty-two, and some children were 4 years old, 5, or 13. I’m truly ashamed to admit it; I did not like to complain, but as far as I was concerned, I would have preferred to be there with my mother, holding on to her, almost crying. But in the end, I would say to myself: “Behave yourself!” (P2).

### 4.3 Theme 3: musical anxiety factors

#### 4.3.1 Factors that reduce anxiety

These interviews showed that our informants’ families were the principal factors that helped reduce their anxiety, providing an immense amount of support in critical moments. “My father helped me a lot to study [my instrument]” (Vn); “My parents helped me in every aspect even though they do not have any knowledge of music” (P1); “I think that those who have helped me the most have been my mother and my father. Sometimes, I tell them that [university-level] conservatory has me down, that I’m not coping well, and that I’m not progressing. It is they who motivate and pick me up the most” (Vc); “My mother has helped me a great deal; she has done relaxation [therapy] on me” (P2).

Teachers who show emotional empathy with their students and display a positive attitude toward them have also been an anxiety-reducing factor. “My teacher also raised my self-esteem; he was something like a father” (Vn); “My teacher… I’ve had such great luck... I believe that with another teacher I would not have managed to finish my studies... I remember I had [female] friends who would come crying out of the classroom due to the level of expectation and requirement. But my teacher knows how to incorporate emotional intelligence into music, and they have helped me a lot …” (P2); “Another thing that has helped me is when teachers recount experiences they had” (Vc); “I had a teacher who had good mental health; in other words, he really enjoyed what he was doing. Everything, for him, was [done] for the sake of enjoyment and improvement.” (Vn).

Playing music with other people also reduces anxiety, according to our informants, who reported that they gained much enjoyment from such experiences:

I always enjoy music more when I make it with others. That’s something obvious to me. And generally, I prefer small groups, simply because it’s what I like the most … (Vn).

I got the most enjoyment from chamber music; for instance, I played piano with a [female] violist and a [female] singer. That’s where I enjoyed making music the most, as the focus of attention was not just me; the responsibility was not mine. I thus knew that if I made a mistake, my two friends would be there to forge onward, and I would find my way back into the music. That’s where I’ve really enjoyed it a lot. I’ve also enjoyed singing in church choirs; I’ve enjoyed it like no one else could. I also like it so much when the audience applauds… that’s the top, the utmost. (P2).

Whenever I’m [playing] in a group, I do not tend to feel any anxiety because I feel that my colleagues are covering me. The moment where I most enjoy [making music] is when I play in the orchestra. (Vc).

In one particular case, the act of improvisation appears as a factor capable of reducing anxiety:

When I improvise, I do not feel any sort of anxiety whatsoever, as any mistake can be transformed to become part of the improvisation. Particularly since perfection does not exist in improvisation, it simply does not exist! If nothing has been set out beforehand, no one can say that what you are playing is incorrect, in a way of speaking. On the contrary, in classical music, we are interpreters: we are supposed to interpret what’s indicated in the score, that which has been stipulated, what we are supposed to do, respecting the style … I thus feel that all these types of things are what creates the greatest amount of tension when you are about to play. (P1).

However, another informant suggested that she did not make progress in improvisation because it had not been included as a subject in conservatory training from the beginning:

Currently, improvisation is only featured as a subject in the last two years of the conservatory curriculum. So, sure, let us just start improvising when we are twenty! I do not see the point at all; if anything, at twenty, you are cognitively more rigid, and people like me, who suffer from anxiety… I just find that… Or, let us put it another way: What do you want me to improvise [for the first time] when I’m twenty? Until then, no one had ever taught me how to improvise, not for a moment, not even for ten minutes. In other words, improvisation is something that needs to be taught: there are mental patterns and chord patterns… In other words, improvisation does not just mean that you sit down and start to play. So, definitely no, that’s a facet I have not been able to develop. No. I’ve given up improvising. (P2).

Nor do conservatories feature a subject in their curriculum devoted to relaxation and designed to help students reduce anxiety, as this interviewee points out:

The truth is, I do not know how to control my nerves before a recital. Neither am I familiar with any sort of relaxation technique; I do not know what I could do before performing a recital: there’s got to be some way of relaxing oneself, or a thought you can have to motivate yourself and do it well and not feel so much anxiety. But I do not know any of them. They could offer a course in conservatory, even if it were not obligatory: just 1 h a week, on how to relax and confront recital situations. Yes, it would help a great deal; it could have helped me a lot, too. (Vc).

#### 4.3.2 Factors that promote anxiety

On the other hand, on occasion, teachers can play a role that *promotes* anxiety, particularly those who teach at advanced levels. The following case relates how a composition professor lowered a student’s self-esteem with comments that were not particularly constructive:

I had a composition teacher when I entered Professional [secondary level] Conservatory. I also tell this story a lot. It’s about... a composition teacher who, on the first day, when I was a little girl, I wanted to be a composer. And the first composition he made us do [as homework], I was there at home, enjoying it a lot [by trying it out on] the piano; I [then] brought him my composition [to class]; I must have been 14–15 years old. And well, he basically told me that it was a piece of shit, that I could throw it away. Of course, that was a big shock for me, since I liked it. And that’s when I put up a lot of barriers. And it took me a long time to say to myself again, hey, I’m going to try it again. Because I assumed from that moment on that I wasn’t good at it and that was it. So, of course, you have to be very careful, because certain ages are vulnerable, and well, regardless of age, it’s something you should not do [no teacher should do to a student]. Because... simply, he also made that remark because I had written something modal. Back then, I did not even know what “modal” was, and there was no tonal tension in my piece, right? And then he told me that it wasn’t... that it wasn’t valid. And that was it. And I remember that teacher many times, and of course the recollection is unpleasant... And I have not gone back to composing, except for some meagre attempt, but well, it’s still there, eh? Yes, yes, that [nagging feeling of] “I do not know if I’m good at this or not.” (Vn).

Another of the four respondents provided the following reflection regarding a general lack of pedagogy in music teachers:

The problem is that the teachers all have a violin, and they are violinists or pianists... But they have been with a student weekly for 10 years, and that’s all they care about. There are many more things... of course. And I have talked about it with many teachers. The lack of knowledge about pedagogy seems to me like a basic flaw, you know? You have to know pedagogy; you have to know it. You need to have a certain degree of psychology, right? Something, at least something. Learning, education, contact with families, tutoring with families... (P2).

Two of our four respondents mentioned the requirement of playing pieces by heart:

I principally associate [performance anxiety] with the requirement of having to play by memory. That is what usually leads me to have that anxiety. The more I have to play by heart, the more I observe that my anxiety grows because I am even more afraid of making a mistake. Because I’m worried that my memory might fail me and I might go blank in the middle of a performance. (Vc).

Learning by heart… I just could not deal with the aspect of memorizing … (P2).

### 4.4 Theme 4: personality traits

Perfectionism and considerable demands on self were two likewise notable characteristics, along with fear of failure or fear of disappointing others, whether performing alone or in a group:

For me, the issue is that the performance is supposed to be at a perfect level, and you have to play a score exactly as written. You cannot skip anything, you cannot. And if you make a mistake, it’s a failure. (Vn).

The more people I depend on, the more that factor [of anxiety] appears: that fear of “not making it.” How can I explain? When there’s something – not just in music – that depends exclusively on me, perhaps I may achieve it more or less well, but I’m still confident that it only depends on me. However, if there’s something where I depend on a greater number of people and I have to become more organized, it makes me afraid: that very thing, the fact that it depends on more people. Not so much the possibility that they might make a mistake or whatever, but, rather, that I could make a mistake, and that, because of my mistake, I might ruin it for them, and the entire concert might be ruined. (P1).

Last year, I played a chamber music piece with a classmate who played the piano, and our result was terrible compared to how it sounded in class. And I felt a little bad because it was mostly my problem, and I was the one who was the most nervous. I was playing with a classmate and felt a little bad for him because I obviously did not want to give him a hard time either. Also, because I did not want to “disappoint” our teacher, so to speak. When I have a concert coming up, I always think about what the teachers will think when I’m done. If they will have liked it, or whether they will have found that in the last weeks I have not studied as much as they had asked me to. So, always, the question of what the teachers’ expectations will be is one of the things that influences me the most. (Vc).

One of our four respondents defined herself as a perfectionist, as someone who is anxious and suffers from low self-esteem:

Let us see now. I think people who are like me, well, we have that personality of being very perfectionist and anxious. Because I’m anxious about everything, simply about *everything*. There will always be an anxiety component that will not ever go away; to me, that’s self-evident. I think I’ll always be that way. Of course, I also need to learn how to regulate myself. Not to do away with the anxiety entirely. To regulate myself. Because if you think too hard about trying to do away with it entirely, I believe it’s probably counterproductive and you start feeling more anxiety. I’ve never regarded myself as an expert; I’ve never felt on a par with my colleagues. I’ve always found that the others were better than me. Not believing in myself… In this life, we often have to believe in what we are doing, right? If I do not believe in what I’m doing, I cannot enjoy it either, can I? My problem has been that I just have not valued myself enough. (P2).

This person even came to feel guilty regarding her own sister’s anxiety and abandonment of music:

My sister did conservatory at the same time as I did: piano as I did, but now she quit. And I think I’m mostly to blame for that. (P2).

### 4.5 Theme 5: coping strategies

To deal with situations of anxiety, people use different strategies: self-medication, psychological techniques, controlling and selecting food and liquid intake, or actively applying a capacity for self-criticism attained after years of professional experience.

#### 4.5.1 Self-medication

The two pianists in our cohort affirmed that they had taken Sumial, a beta-blocker, to reverse the physical effects caused by anxiety.

I’ve taken Sumial quite a few times. I started taking it in superior [university-level] conservatory, in those moments in which I could no longer allow that tremor in the hands that did not allow me to play. I also have to insist that I naturally have a bad pulse, so imagine what starts to happen when I go on stage when I have to start to play; of course, the trembling starts. In those moments, I could not allow my hands to tremble: everything had to be in place; moreover, certain goals had to be met. So, the moment had arrived [to take a beta-blocker]. (P1).

I could not play a super-rapid passage with my hands like that: frozen, sweating, everything [on the keyboard] was just slipping and sliding. That’s when I started taking Sumial, which, as you probably know, is making the rounds in conservatories – whether for better or for worse, I do not know. I was able to get some without a prescription. I was able to get a package, and the result was marvelous. Why? Admittedly, the *thoughts* I had were not dispelled by taking the pill, but I managed to control the entire physiological aspect. My heart rate slowed down; my hands no longer sweated, and I could control my foot [which controlled the pedal]. (P2).

#### 4.5.2 Self-therapy

One of our respondents recounted that she had used cognitive analysis strategies to deal with the stress of a university-level music academy audition. In that particular case, she attempted to confront her anxiety by adopting a logical view of her situation while recalling positive emotional reinforcement moments and trying to avoid focusing on mistakes:

I tried to analyze the “reason why” a little bit. Thanks to that, every time I went out [in front of a jury], I tried to play down the importance of the fact that it was an examination. I tried to tell myself that this was not in order to get a grade; it was because I liked it, and I wanted people to enjoy what I was doing. But of course, I had to repeat this a lot to myself. And, of course, I also remembered many of my childhood moments when my parents, my sister, and my teacher were there when I played, usually congratulating me no matter what occurred. [I did this] to [try and] get rid of the burdens and weights that this musical training has, and to gradually start ascribing less importance to things that are actually not important at all but which, for some reason, are a burden for me. Think, for instance, colleagues who are probably saying to themselves, “Look how well she’s achieving that,” but in your head, you see a “wow, they are seeing that this or the other thing has gone wrong,” and you focus on the mistakes, do not you? Well, I can do a little bit of self-therapy [consisting of saying to myself], “No, focus on the good things, and stop viewing other things as important when they aren’t.” (Vn).

#### 4.5.3 Learning to visualize

Another respondent used mental visualization, a technique employed in psychological therapy to reduce stress and anxiety, in an attempt to bring the mind and the body into a profound state of relaxation:

My mother’s colleague really helped me learn to visualize the situation. Lying in my bed, I imagined myself going out on stage: how I was going to enter the stage, to have it all more or less under control, to know what I was going to do, and to know how I was going to wrap it up. The most important thing was to know how to deal with the situation and the reigning mood, not to let them overcome me. (P2).

#### 4.5.4 Nutrition and reducing stimulants

Informant P1 found that appropriate nutrition is one way of dealing with anxiety: in his case, he ate bananas before each recital.

Although I have never liked bananas, still, before recitals... I still eat two bananas. [Prior to concerts I had to give], I used to eat bananas like crazy, well, because of the [nutritional] content and, well, yes, the truth is, they are one of the things that have had an impact, things that have helped me to fight that anxiety. (P1).

Another interviewee indicated that she avoided stimulants. “In the days before the recital, I do not drink coffee; [I do not drink] anything stimulating that might accelerate my heartbeat.” (Vn).

#### 4.5.5 Professional trajectory

For Informant P1, the fact of having obtained his music diploma and finished his studies makes him feel more secure in the face of anxiety, as well as the fact of no longer being judged by a jury:

Over the years, after finishing the Superior [university-level] Conservatory, I have started becoming more indifferent to things while at the same time becoming more self-critical. I have cared less about what outsiders think and have become more critical of myself. I have gained confidence, which has also allowed me to evolve technically and musically. I trained toward a Master’s degree in performance with different teachers; in this case, I think I had 5 or 6 different professors. What was interesting at that level was that I could attend all the other students’ one-on-one classes as an auditor apart from receiving my own individual lessons. And between everything I saw, everything I heard, and everything I received in those classes that did not have such an “evaluative” factor, perhaps it all allowed me to become a little more relaxed and to be a little more concentrated in analyzing each aspect of what was being interpreted, of what was being corrected, either to another student or to myself, to... well that’s it, in a nutshell: to be able to be more relaxed. (P1).

### 4.6 Theme 6: the approach to dropping out of music studies

Two of our four interviewees did not abandon their studies but went on to university-level music academies. The remaining two finished what in Spain is called *Grado Profesional*, a pre-university-level music diploma. Regardless, all four contemplated dropping out at one moment or another of their careers. If two of them decided to go on, they ascribe their motives to the effort they had already invested over all those years, as well as to the twofold challenge – personal and professional – of constantly attempting to improve, thereby gaining in terms of self-knowledge.

I’ve always had that duality of “I do not know if I want to go on with this.” Because there undoubtedly are moments when it’s a little hard, in which you ask yourself: “I do not know if it’s worth the trouble to fight so hard,” but then there are other moments that have compensated for those. Clearly, putting them in balance has led me to go on. Moreover, it’s helped me know myself better and improve in other aspects. (Vn).

Yes, there have been moments in which I envisioned quitting, precisely because something did not turn out well for me, and I thought that perhaps I might not be able to continue. Then, obviously, if I started to sense that everything could collapse, everything I’d been fighting for all those years, then, as far as I’m concerned, my response is: “Never!” (P1).

I managed to finish *Grado Profesional* [secondary-level conservatory]: out of self-respect, I suppose, but I was overwhelmed by the situation and decided not to go on to *Grado Superior* [university-level music academy]. (P2).

Yes, I thought [of dropping out] in the fourth year of elementary music education, with all the stress associated with trying to pass the exam to *Grado Profesional* [secondary-level music education]; I did indeed contemplate the possibility. But usually, whenever I thought of dropping out, those were just temporary musings. Thus, I have to say, yes: I see this as a problem, a personal problem. I would certainly like to feel better after concerts. Right now, I do not know if I could even imagine trying to earn a living from music, nor would I ever consider studying *Grado Superior* [university level]. Of course, I’ve always loved music, but from now on, I want to have it as an accessory: a hobby. For instance, I could play once in a while in an orchestra, but just to have fun. And admittedly, the subject of stage fright tends to discourage us a lot: a professional musician plays in front of hundreds, even thousands of people, and accomplishes it with perfection. But no matter how much I practice and improve, I do not know if I will ever be capable of achieving something like that. Stage fright truly does pull me back. (Vc).

Anxiety can be partially overcome with the passage of time, as in the case of the following informant:

I would say that although I have overcome it, it’s still always there. It’s always there. True, but if it enters my mind, I try to dispel it. (Vn).

One of our respondents reported that she continued to suffer from anxiety to the extreme of feeling an aversion to her instrument:

As chance would have it, I have moved to a new place, and the piano has remained in my mother’s home. But even before, with the piano in my own home for 2 years, I did not touch it. It gives me… the creeps. It caused a certain amount of anxiety, still today. Currently, I can say that I have an aversion to the piano. When I see a piano somewhere, and I just have to leave the room. As soon as I see a piano, my heart starts pounding “Boom Boom Boom.” (P2).

### 4.7 Theme 7: strategies to help students

Three of the four musicians we interviewed are currently in the teaching profession. After their experiences with anxiety, they state that they are trying to spare their students a similar experience. Certain strategies with a potential for helping students to successfully avoid anxiety are the following:

#### 4.7.1 Reinforcing self-esteem

We work on self-esteem techniques in class during the entire course. When the moment of the recital draws near, already a couple of weeks earlier, the only thing I attempt to do is to boost their morale, particularly in the last week. They have to believe in themselves, they need to have high self-esteem, and they have to play the recital to enjoy it. [I tell them] not to worry if a note does not turn out right. They should do their best, but most of all, they need to enjoy it; I emphasize that they need to feel valued. That’s the most important thing: they should downplay the attitude “If I make a mistake, I cannot go on”; no, it’s all the opposite. (Vn).

#### 4.7.2 Focusing on objectives

I think it is possible to teach in a somewhat healthier manner. To focus on the goal, not on the technical aspect. Of course, the technical aspect is there and has to be there, but it is not the ultimate goal; it is a means to achieve it. I think if we gave more importance to music in itself, then less people would quit. But let us be clear now: I think many people abandon their music careers because of the teachers they have. That’s something eminently important. If the teachers are hammering you – just as in any other discipline such as sports, dance, and artistic discipline – if they hammer you only with technique, you end up wondering: why? Why so much effort? What’s the point if, ultimately, there will always be something you do wrong, and there will always be someone better than you? Moreover, they train you a lot to be a soloist, but people tend to forget the [important role] of chamber music, playing in a group, enjoying playing with others. I thus think that is important. (Vn).

#### 4.7.3 Improvising

I have them [my students] improvise a lot from the beginning to ensure they are not afraid of it. Admittedly, you’ll find some children that just do not have the knack, but I’ve seen in children from age six on, when you say: “Come on, take the violin and play whatever you want,” and you see their face all tense and blocked, as if they were saying: “What? No way. If there’s nothing in front of me, how am I going to…?” And you feel like telling them: “But you are only six!” and you think: If you do not have imagination now, pffff, things will just get worse later. (Vn).

#### 4.7.4 Encouraging creativity

By making a comparison between musical learning and language learning, one of our informants pointed out a general lack of creativity in music education:

If I have to do everything perfectly [as written in the score], I cannot skip anything; I have to pay attention and ensure I do not skip over any of the 200,000 notes on those sheets. That leaves creativity far behind. Because no, no... those notes do not give you much room to play around in. You are so concerned about technique, trying to make sure you do not skip over anything, that everything turns out perfect just like you studied it, to the best of your abilities. Thus, of course, creativity is gradually erased, it is eradicated because you never work on it. I try to describe the situation a little bit like this: if a child who was supposed to learn to speak learned to read and write before learning to talk, what would happen? They would spend their whole life reading and writing, just reading, writing, but they would never speak using their own language. So, obviously, if you do not work on creativity, it gradually fades away. (Vn).

The same person stated that creativity helped them in their quest for self-knowledge, and it should also prevent children from falling into situations of anxiety, as it additionally helps them better understand music:

Creativity has always immensely helped me to get to know myself. Thus, if I want to pass [creativity] on to my students, I have to work on it myself, given that I do not have criteria that allow me to say, “This or that thing is what helps me encourage creativity”; no, I do not have such criteria; I have to go looking. Creativity allows you to move and play around within that. If you make a mistake at one moment, you can wiggle your way out of it because you are starting to understand what is happening. [When I was a student], I often did not understand what was occurring in music. (Vn).

#### 4.7.5 Adapting the teaching style to each student

Another interviewee who was still active in the teaching profession mentioned that he tried to adapt his music teaching to the needs of his students: “I try to be the best teacher I can be. Obviously, that depends on each one of us. All of this means that I become quite selective when I have to impart a certain type of teaching or another, for example, based on how I view one student or another” (P1).

The only one of the four interviewees who did not give classes pointed out the need for music school staff to include psychologists and other specialists:

Some music schools in Valencia have a psychologist [on their staff]. And I find that fundamental. Just think about the [amount of] human capacity and emotion contained within a music school! It has to be managed one way or another, for example, by offering psychological assistance, well, regardless of what type of psychologist: it can be mindfulness workshops, relaxation workshops, yoga, or having a good physiotherapist. There’s so much to consider. (P2).

#### 4.7.6 Recordings

Recording one’s playing can serve as a strategy for instrumental improvement, but recordings are not directly related to anxiety reduction. Occasionally, the result can even be disappointing:

Recording myself used to be more difficult [for me]: of course, you listen to yourself and think: “Oh my God!” But each time it’s less difficult. Now, I enjoy it; I listen to myself, and it helps me improve on things I’m unaware of while playing. (Vn).

My teacher has said that recording oneself is a good exercise, but the truth is, I do not usually do so when I play alone. Sometimes we record ourselves when [we] play chamber music in a group, when we do something together as an ensemble. The difference is clearly noticeable and yes, it’s a way of learning. Especially regarding the aspect of intonation, there is no comparison between when you are playing by yourself and when you hear yourself [on a recording]; it can be a bit disappointing, to be honest, when you listen to yourself on a recording because you thought it sounded much better than it actually did. I find that the sound quality and intonation are better when I hear myself playing live than when I record myself. (Vc).

## 5 Discussion and conclusions

Our study’s general goal was to gain an improved grasp of subjective experience in relation to MPA, as related by musicians who have suffered from it and who have had recurrent thoughts of abandonment, which have even materialised. It is also believed that this study complements and fills a gap in previous research.

Our interviewees’ personal history, linked with their musical upbringing and training, included a series of positive experiences as well as certain moments of anxiety. In all these, the family played a fundamental role, particularly during the first years of musical training (Creech, 2010; Oliveira et al., 2021).

Our respondents experienced a greater number of gratifying moments in their first years of contact with music, particularly during elementary music training and education. At that stage, they recalled pleasant moments in class, on the one hand, as well as in recitals or auditions, on the other (Casanova et al., 2018; Osborne and Kenny, 2008; Zarza-Alzugaray et al., 2018). Perhaps because, in their childhood, they were less aware of the learning process and perceived it as a game, or because people tend to accumulate more negative experiences over time (Osborne et al., 2005; Urruzola and Bernaras, 2020). Highlights among those positive situations included the experience of playing in a group, which created an emotional connection among its members (Robson and Kenny, 2017). Interpreting non-classical repertoire can also lead to pleasant, entertaining experiences, encouraging a deeper emotional bond with classmates (Perdomo-Guevara, 2014).

As musicians advance in their training and perfect their technique, they begin to face situations of anxiety (Dempsey and Comeau, 2019; Papageorgi, 2022; Patston and Osborne, 2016; Urruzola and Bernaras, 2020). Anxiety manifests itself through physical symptoms, including trembling, stomach or abdominal aches, and sweating, particularly in moments when the student feels evaluated, such as conservatory entrance exams, year-end exams, and auditions. The sensation of feeling judged by others makes those moments of anxiety even more intense; conversely, whenever the student does not feel they are being evaluated, they tend to have greater recollections of enjoyment (Coşkun-Şentürk and Çırakoğlu, 2018; Osborne and Kenny, 2008).

Our interviewees found that the most critical and difficult moments were associated with entrance exams to university-level music academy (*Grado Superior*) and instrumental auditions in the last years of their academic studies (Iusca and Dafinoui, 2012; Osborne and Kenny, 2008; Sulun et al., 2018). Pressure not only came from fear of being judged by the jury of teachers but also from comparing themselves with their classmates (Cupido, 2018; Perkins et al., 2017).

Our analysis of those interviews yielded a series of factors that can either reduce or increase MPA. We identified the family as the main reducing factor since the students’ families provided support in pivotal moments (Schneider and Chesky, 2011; Orejudo et al., 2021; Zarza-Alzugaray et al., 2020). Another way to reduce anxiety is by playing in an ensemble with other musicians, which frequently leads to enjoyment.

Improvisation is not taught in the first years of musical training in Spain. Although this hampers the skill’s development, improvisation can still serve as a factor that works in favor of reducing anxiety (Kim, 2005, 2008; Perdomo-Guevara, 2014). Very few music academies include courses on relaxation techniques in their curriculum, but such approaches could also help students decrease their anxiety levels.

Educators also play a significant role in reducing or increasing anxiety in their students. Well-prepared educators who are familiar with coping strategies, who commit themselves on an emotional level, and who attempt to remain positive can help reduce anxiety, whereas a professor who is uninvolved and pedagogically deficient (particularly those who teach advanced levels) can increase student anxiety (Papageorgi et al., 2007; Patston, 2014; Ryan et al., 2021). Too much emphasis on playing by rote is another factor interviewees mentioned as a potential trigger for anxiety (Robson and Kenny, 2017).

Perfectionism and self-demand were personality traits shared by all four respondents. Those traits manifest themselves in a constant fear of failure, whether in solo or group performance, leading to an intense sensation of disappointment (Araújo et al., 2017; Coşkun-Şentürk and Çırakoğlu, 2018; Cupido, 2018; Dobos et al., 2019; Liston et al., 2003).

To manage anxiety, our interviewees applied a series of different strategies, including self-medication, psychological techniques, and control of nutrition and liquid ingestion (Blair and van der Sluis, 2022; Burin and Osório, 2017; Fernholz et al., 2019). To reduce stress, they also used mental visualization and cognitive strategies designed to apply a logical approach to the situation by recalling positive experiences while avoiding an excessive focus on mistakes (Burin and Osório, 2017; Oudejans et al., 2017). Some of our respondents resorted to beta-blockers to mitigate the physical effects of anxiety (Studer et al., 2011; Fernholz et al., 2019). The same musicians attempted to adjust their ingestion of foods and liquids, either by eating bananas immediately before a recital or by avoiding coffee in the days leading up to it (Araújo et al., 2017).

All our respondents agreed that anxiety can be partially overcome with time, although it remained present in their lives. Two of our four interviewees obtained a university-level music diploma; the other two obtained a *Grado Profesional* diploma which is prior to university studies. Each one of them, however, considered abandoning their music career entirely at one moment or another; in the study by Orejudo et al. (2021) with Spanish higher education students, the percentage of students with abandoning thinking reached 19% of the sample. Their decision to continue had mainly to do with the amount of effort they had already invested in it, coupled with their desire to achieve self-improvement (Perkins et al., 2017). The accomplishment of having finished their studies reinforced their self-confidence in the face of anxiety, as, once the academic career was over, they no longer felt judged or evaluated. However, these findings refer only to the interviewees and contrast with the results of the study by Kenny et al. (2014), which used a large sample of professional musicians (377 musicians from eight professional orchestras); in this study, self-imposed pressure (with 320 responses) and fear of negative evaluation (with 243 responses) persist to a substantial degree.

Three of our four respondents are currently in the teaching profession. After their experience with anxiety, they now try to prevent their students from experiencing it by applying the following strategies they find helpful: reinforcing students’ self-esteem, remaining focused on goals, adapting their teaching to each student’s individual needs, and generally encouraging improvisation and creativity (Blair and van der Sluis, 2022; Casanova et al., 2018; Iusca and Dafinoui, 2012; Oudejans et al., 2017; Papageorgi et al., 2007; Perkins et al., 2017). Although recordings occasionally produce unsatisfactory results, our respondents find they can generally be helpful in instrumental practice. They also highlight the importance of creating instances of institutional support, such as including a psychologist on the staff of music schools, music conservatories, and music academies (Araújo et al., 2017; Lupiáñez et al., 2022; Perkins et al., 2017).

Although our study has provided valuable insight, it also has potential limitations. The responses to the interview questions were self-reports, thus inherently subjective. It is also possible that the interviewees perceived the researchers as strangers and tended to provide answers on the cautious side. Our sample was obtained by convenience. The number of participants was small, and the sample did not feature a great variety of musical instruments. In the future, to extend the validity of results, information should be gathered from a greater number of informants with more diverse characteristics. Further research lines could include a more in-depth examination of coping strategies, the potential of personalized teaching methods, the role played by the teacher, and an exploration of the first musical experiences of musicians who have suffered from MPA.

At any rate, our qualitative study joins a scant body of MPA literature applying the qualitative methodology: this approach helps us become more familiar with the MPA phenomenon. Our interviewees provided insight into how the subjective experience of MPA is felt and understood on an individual level.

## Data Availability

The original contributions presented in the study are included in the article/supplementary material, further inquiries can be directed to the corresponding author/s.
